# Supplementary figures and images for: A pH‐ and ionic strength‐dependent conformational change in the neck region regulates DNGR‐1 function in dendritic cells
Source: EMBO J. 2016 Oct 17;35(22):2484–97. doi: 10.15252/embj.201694695 (PMC5109244; doi:10.15252/embj.201694695)

A

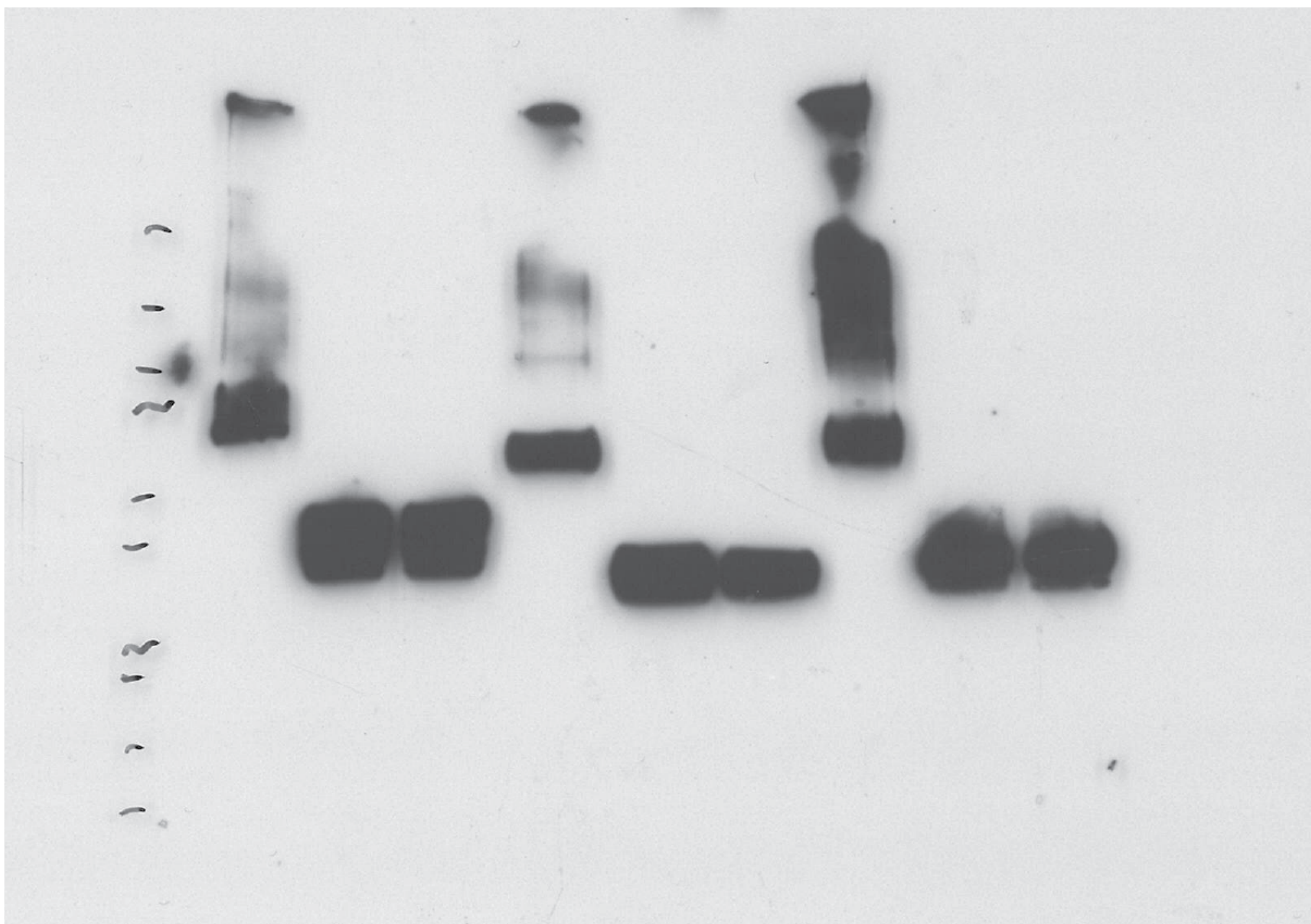

B

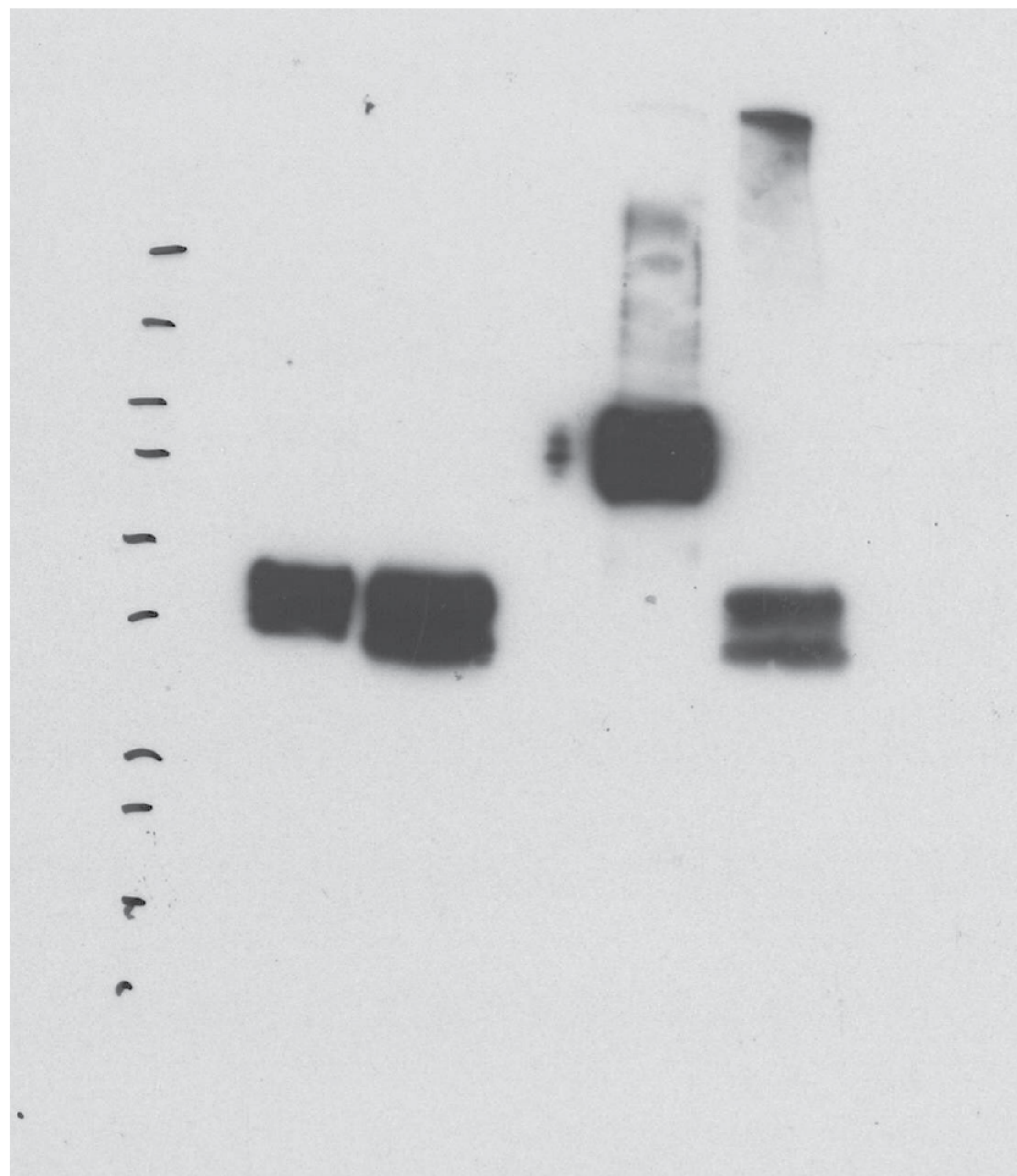

C

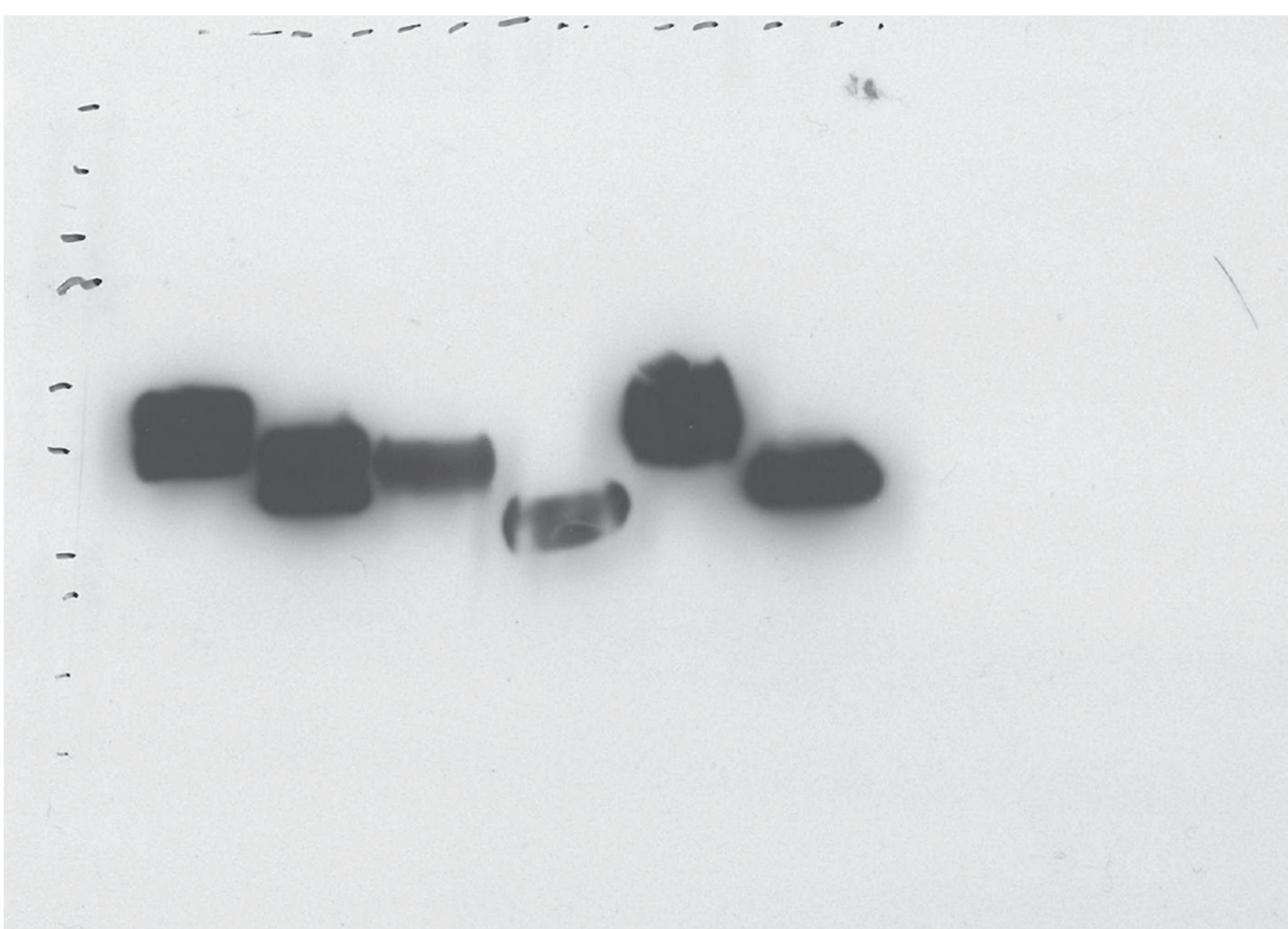

Figure 1 - source data

Supplement: Supplementary file 2 — Source Data for Figure 1 [file EMBJ-35-2484-s001.pdf]

A

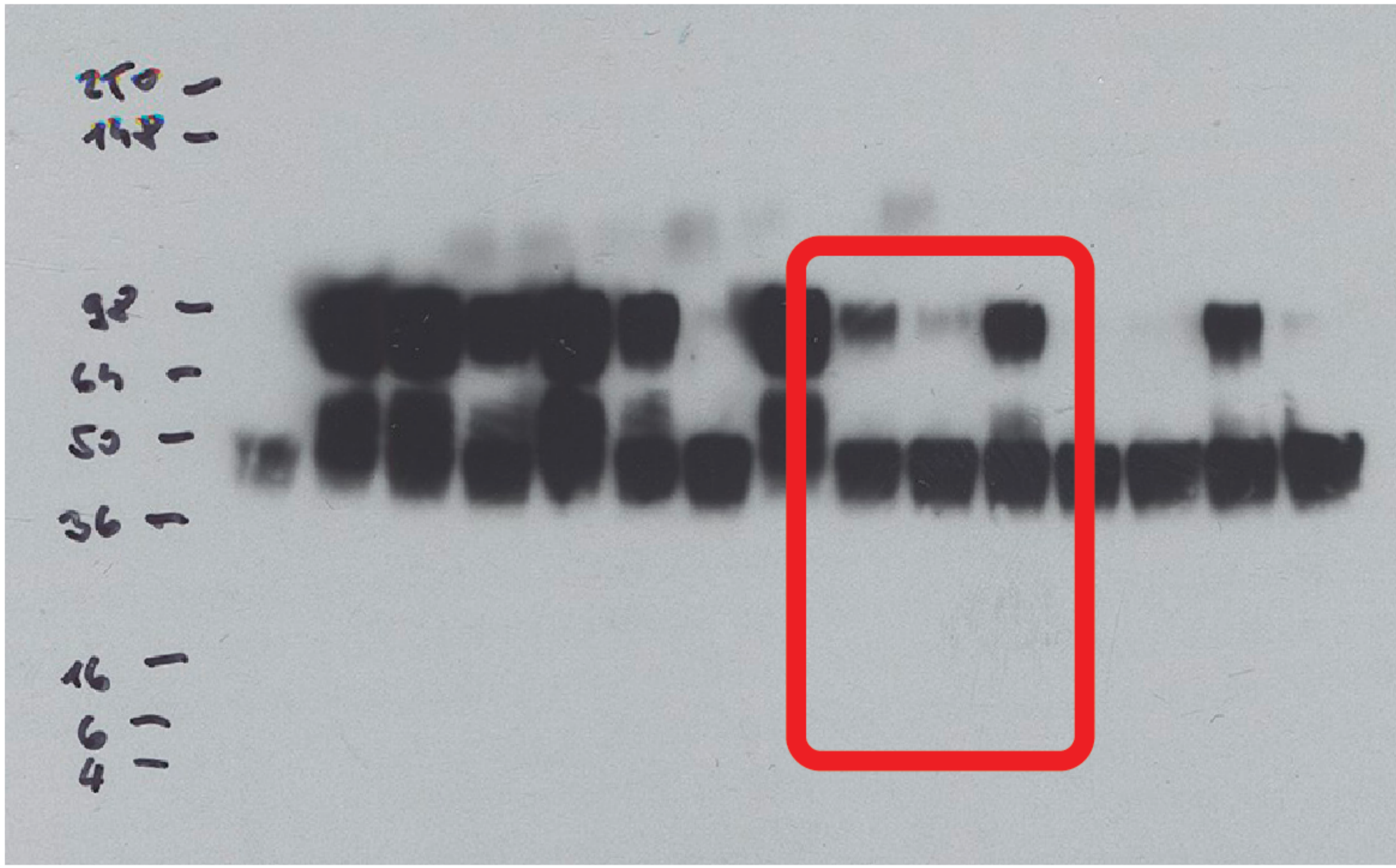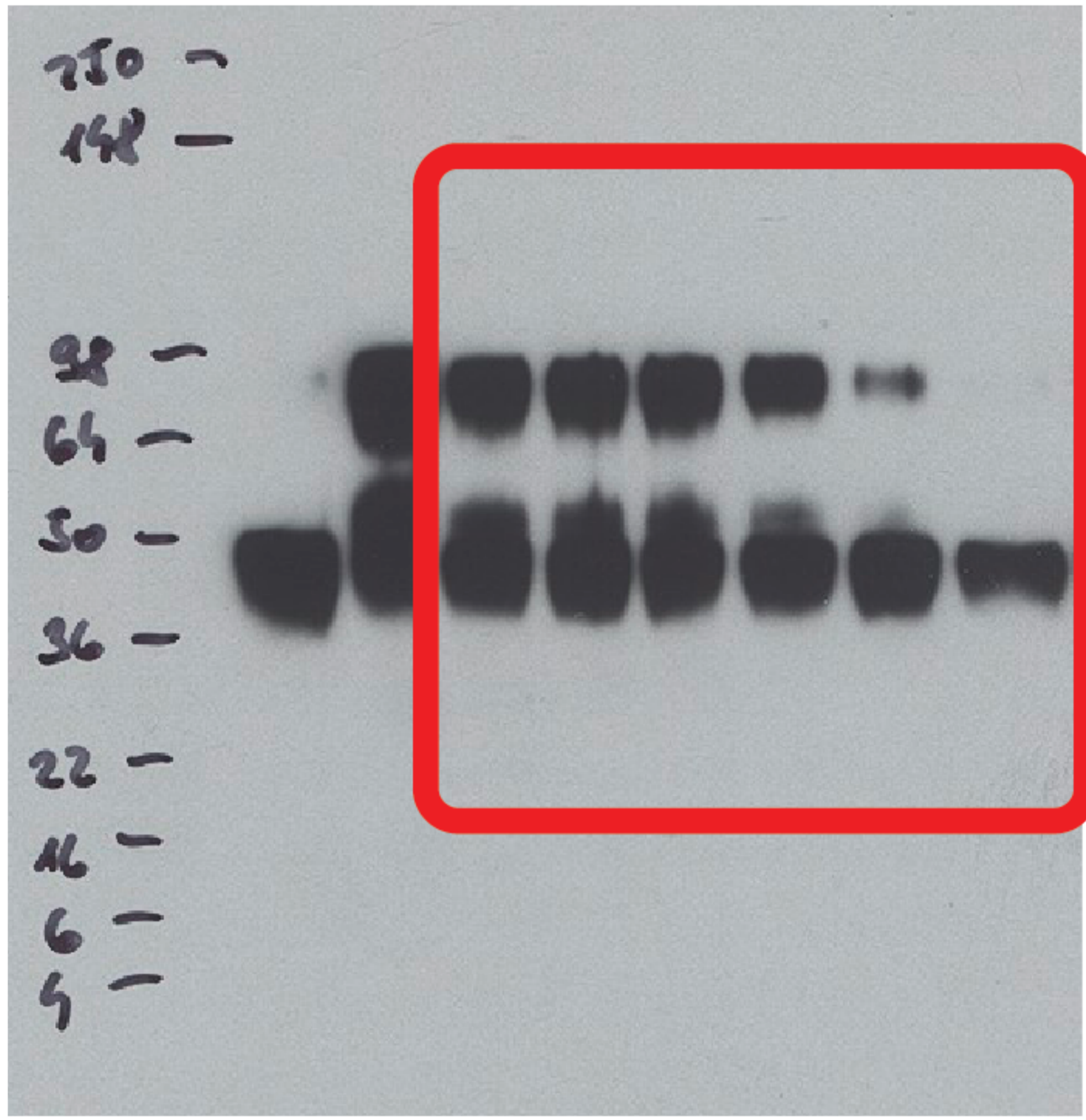

C

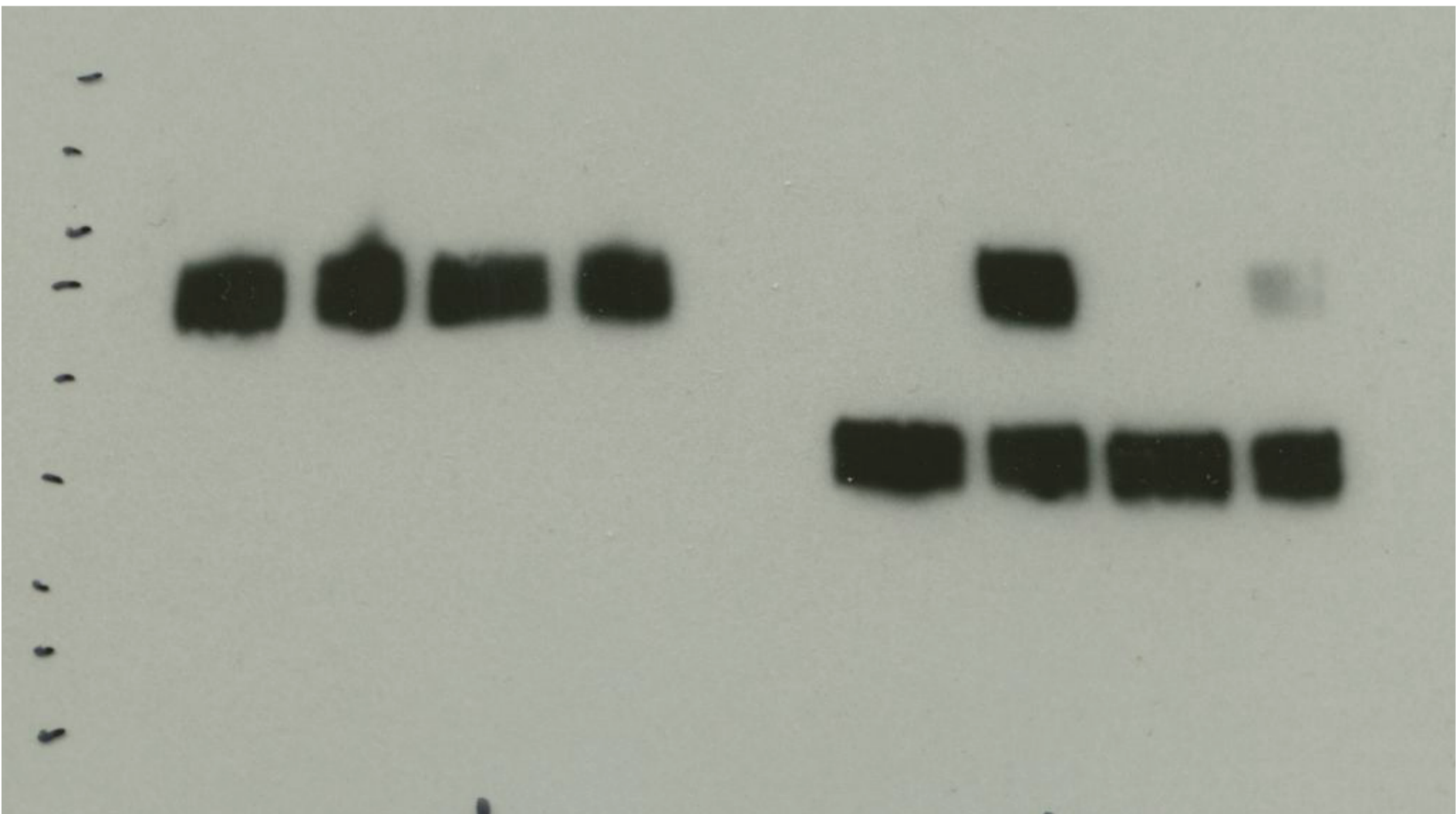

D

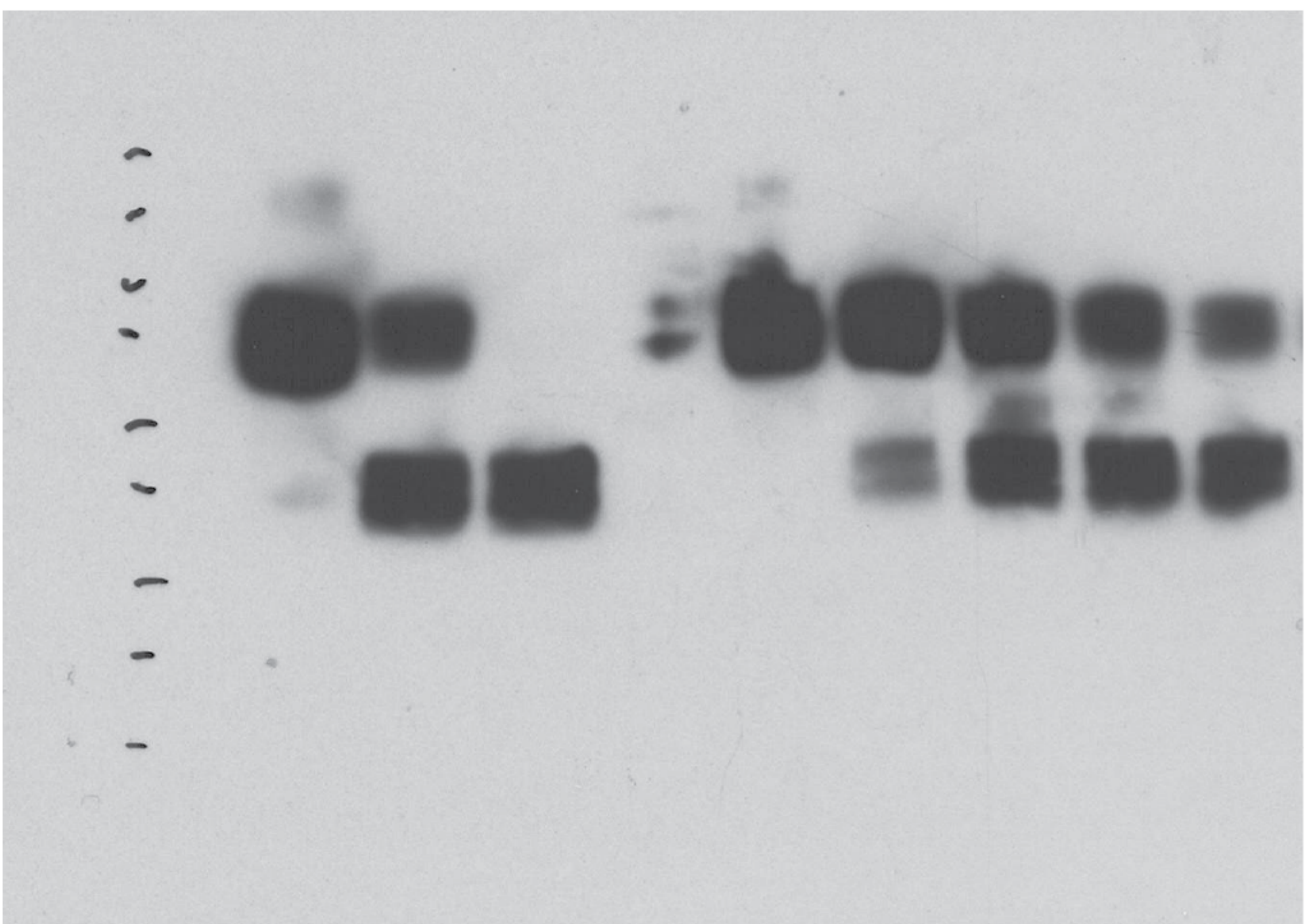

Figure 2 - source data

Supplement: Supplementary file 3 — Source Data for Figure 2 [file EMBJ-35-2484-s002.pdf]

A

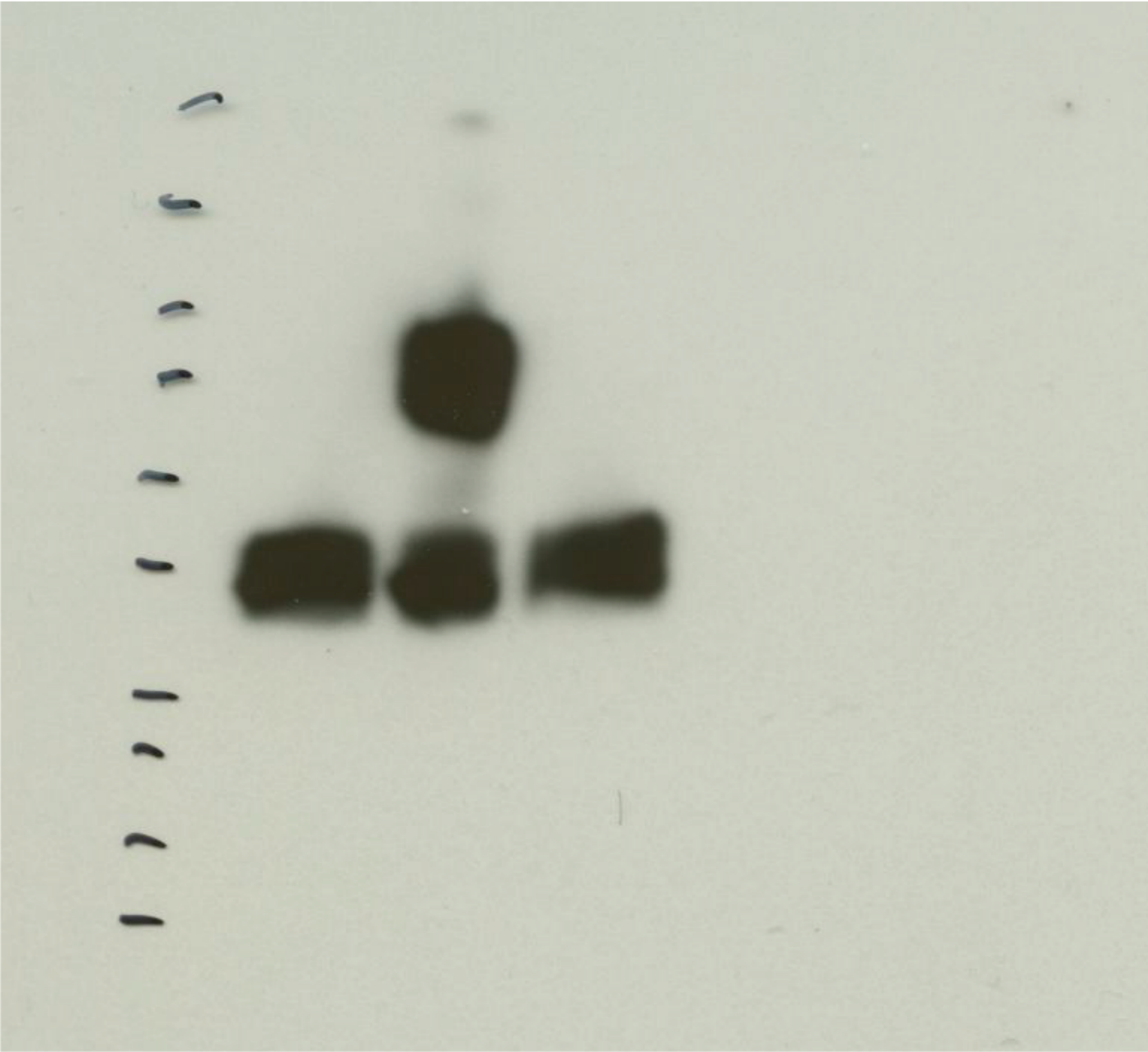

B

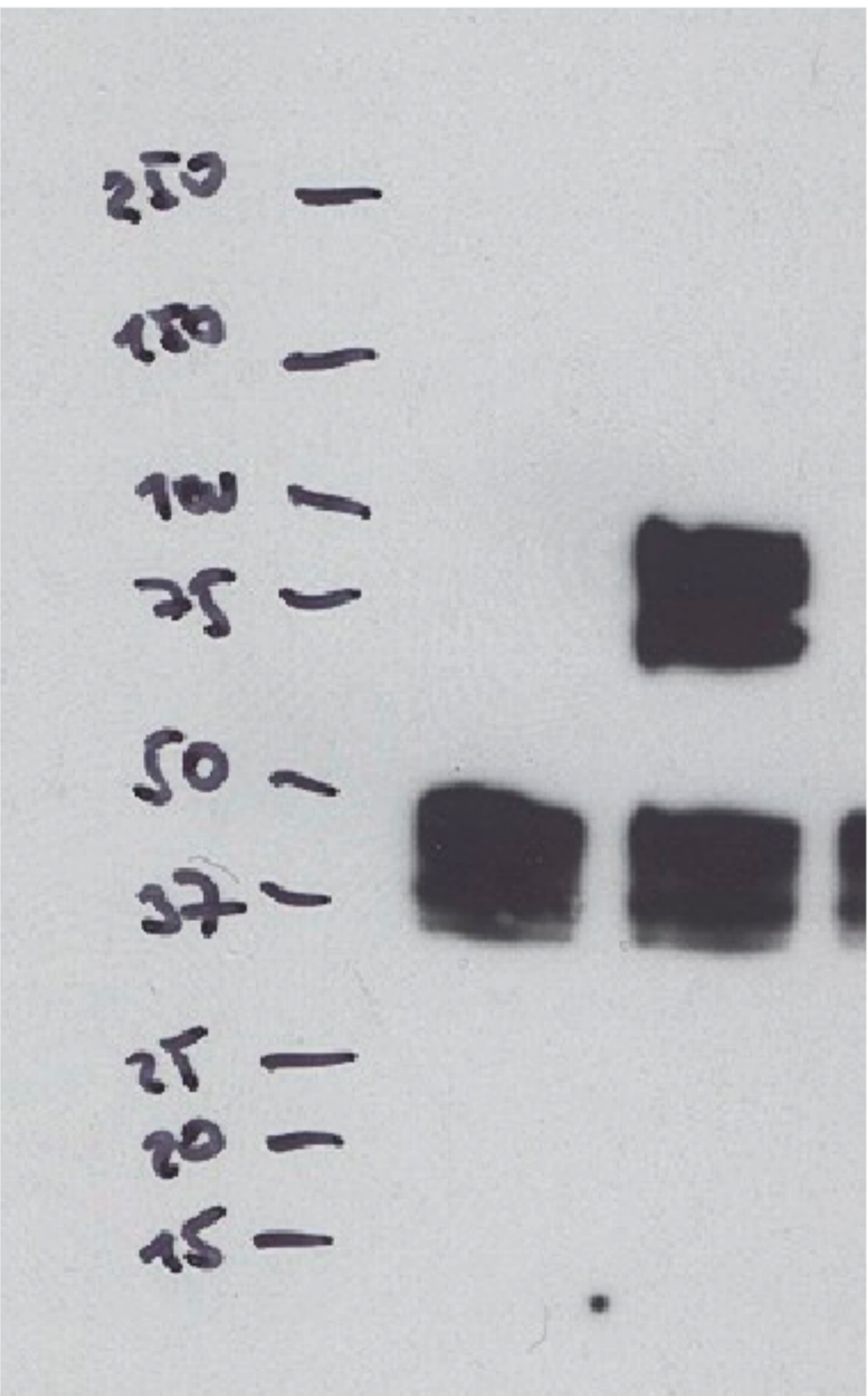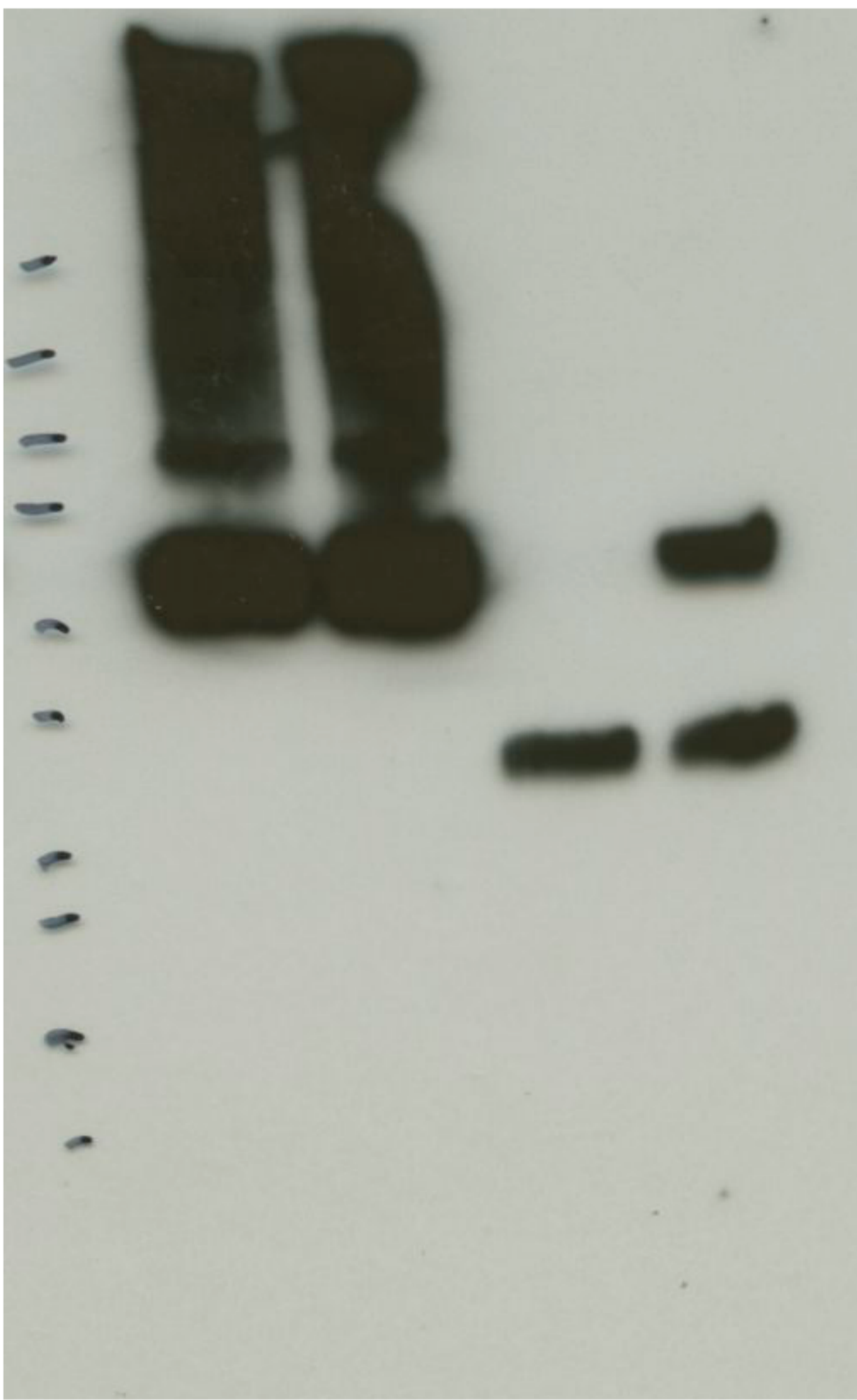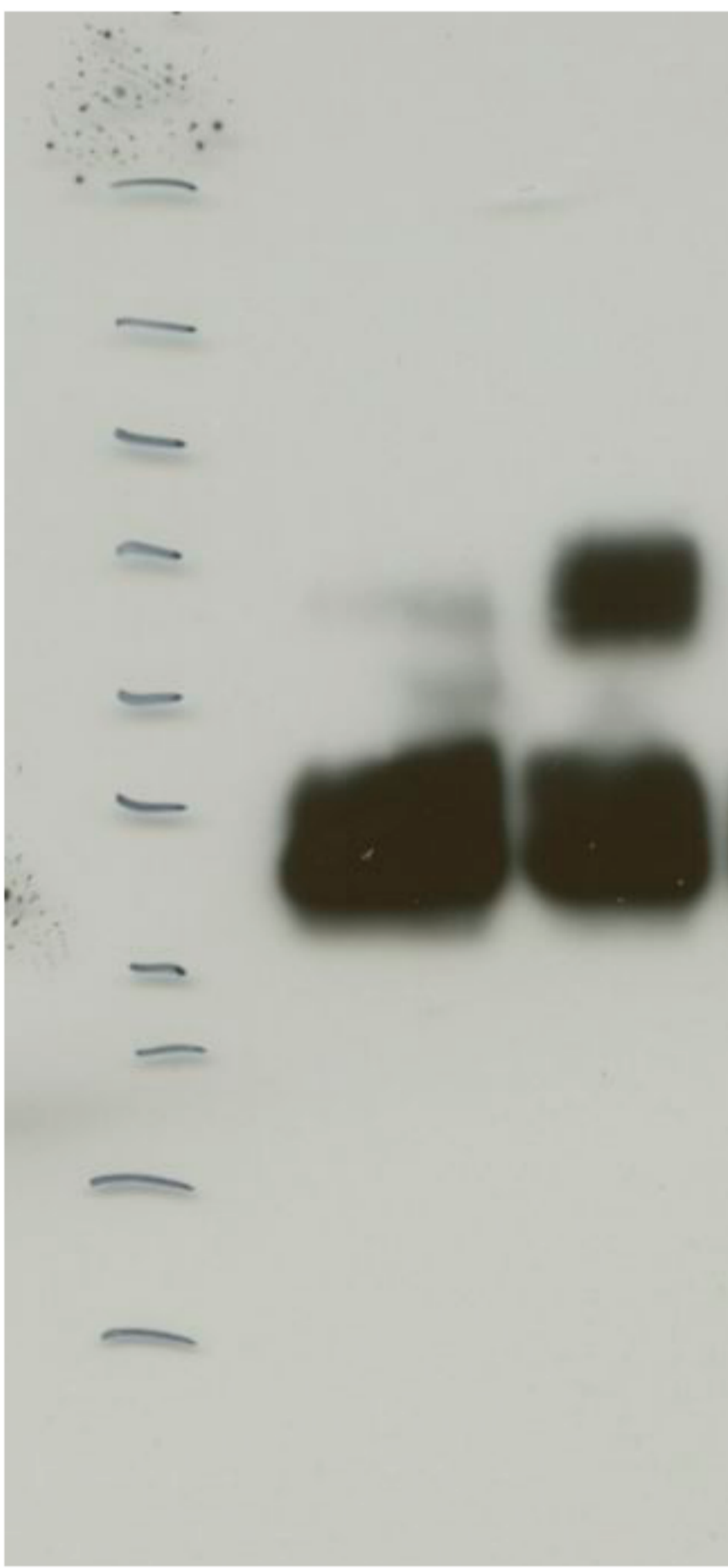

Figure 3 - source data

Supplement: Supplementary file 4 — Source Data for Figure 3 [file EMBJ-35-2484-s003.pdf]

B

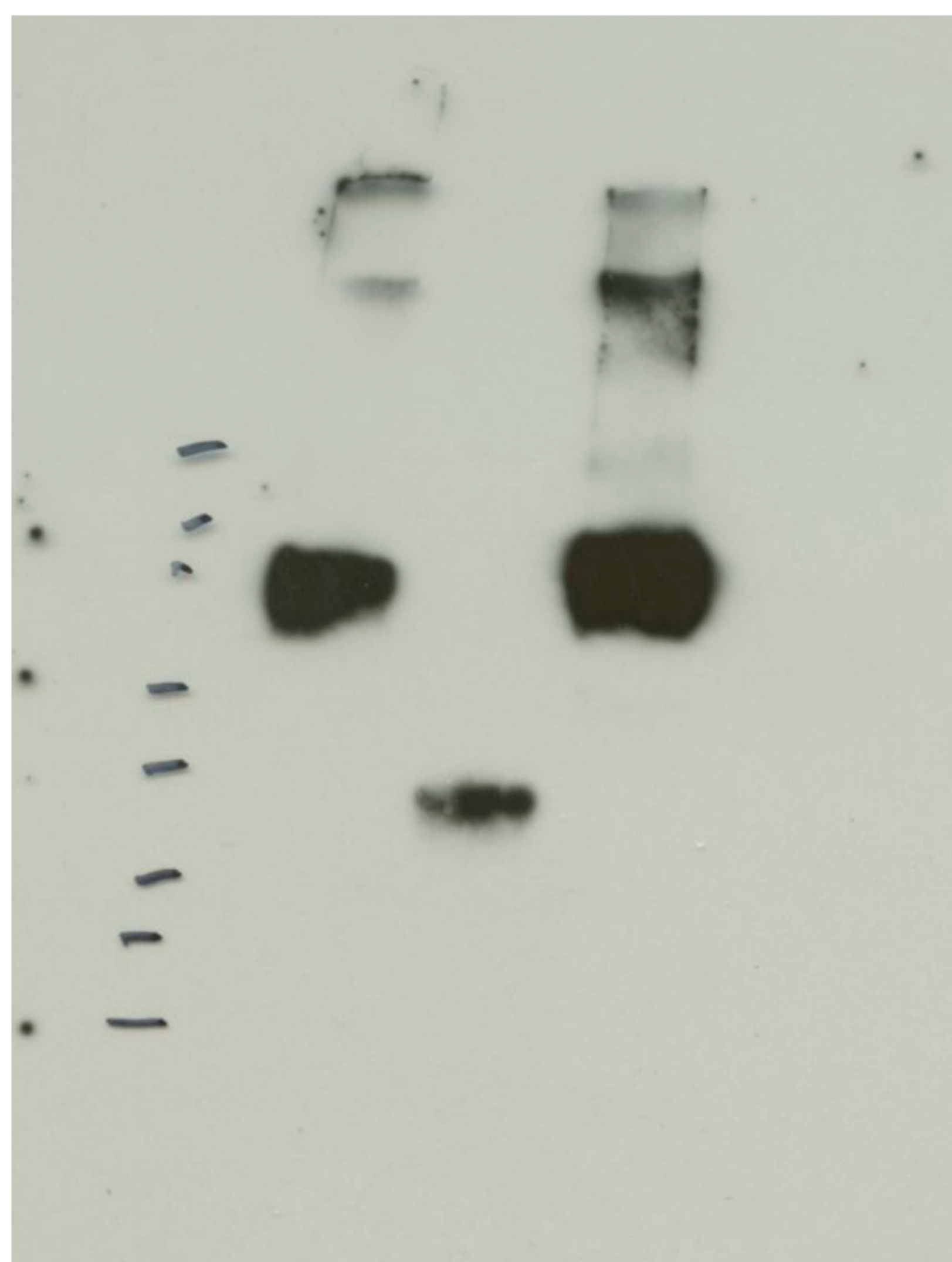

C

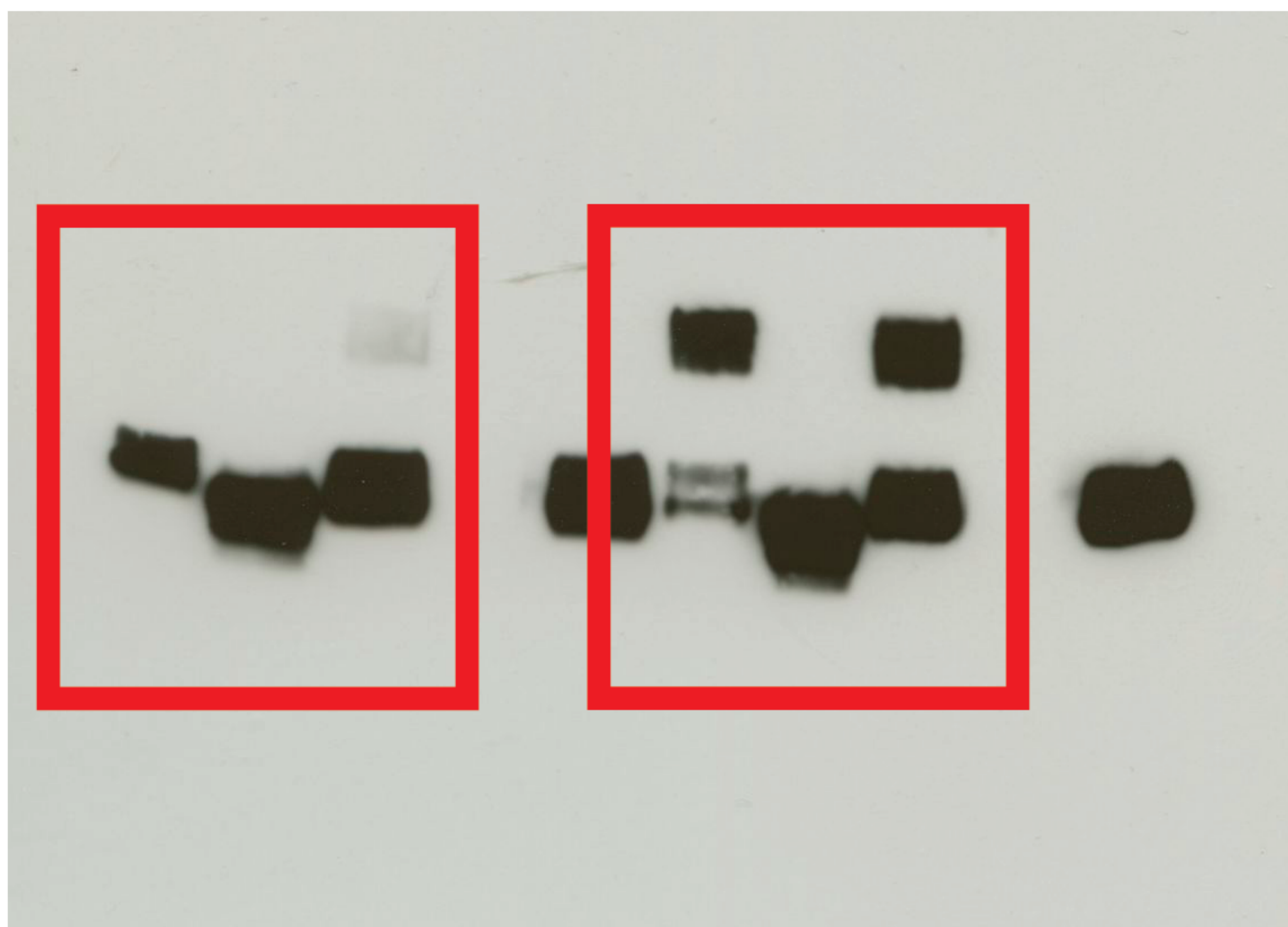

Figure 4 - source data

Supplement: Supplementary file 5 — Source Data for Figure 4 [file EMBJ-35-2484-s004.pdf]

B

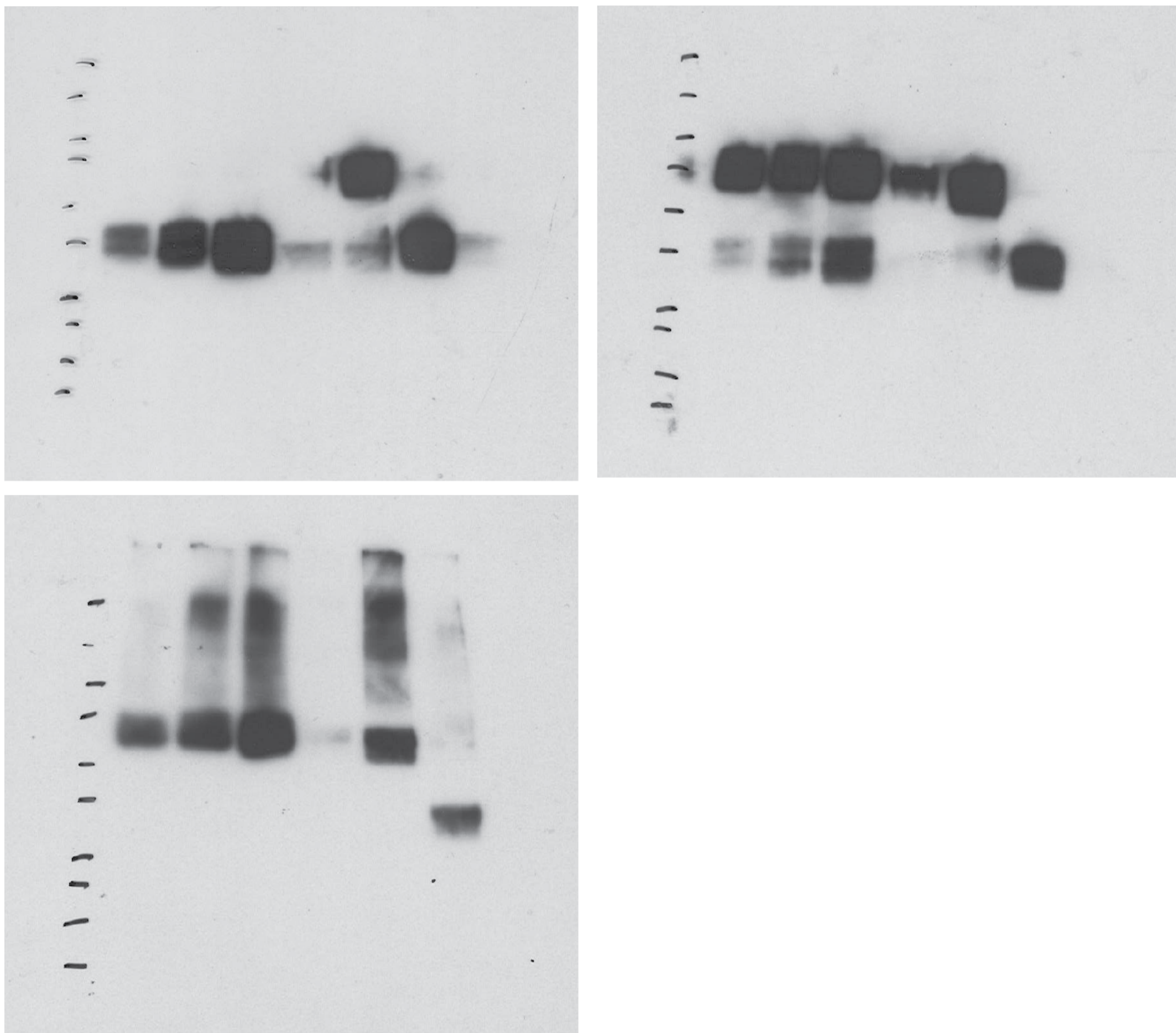

C

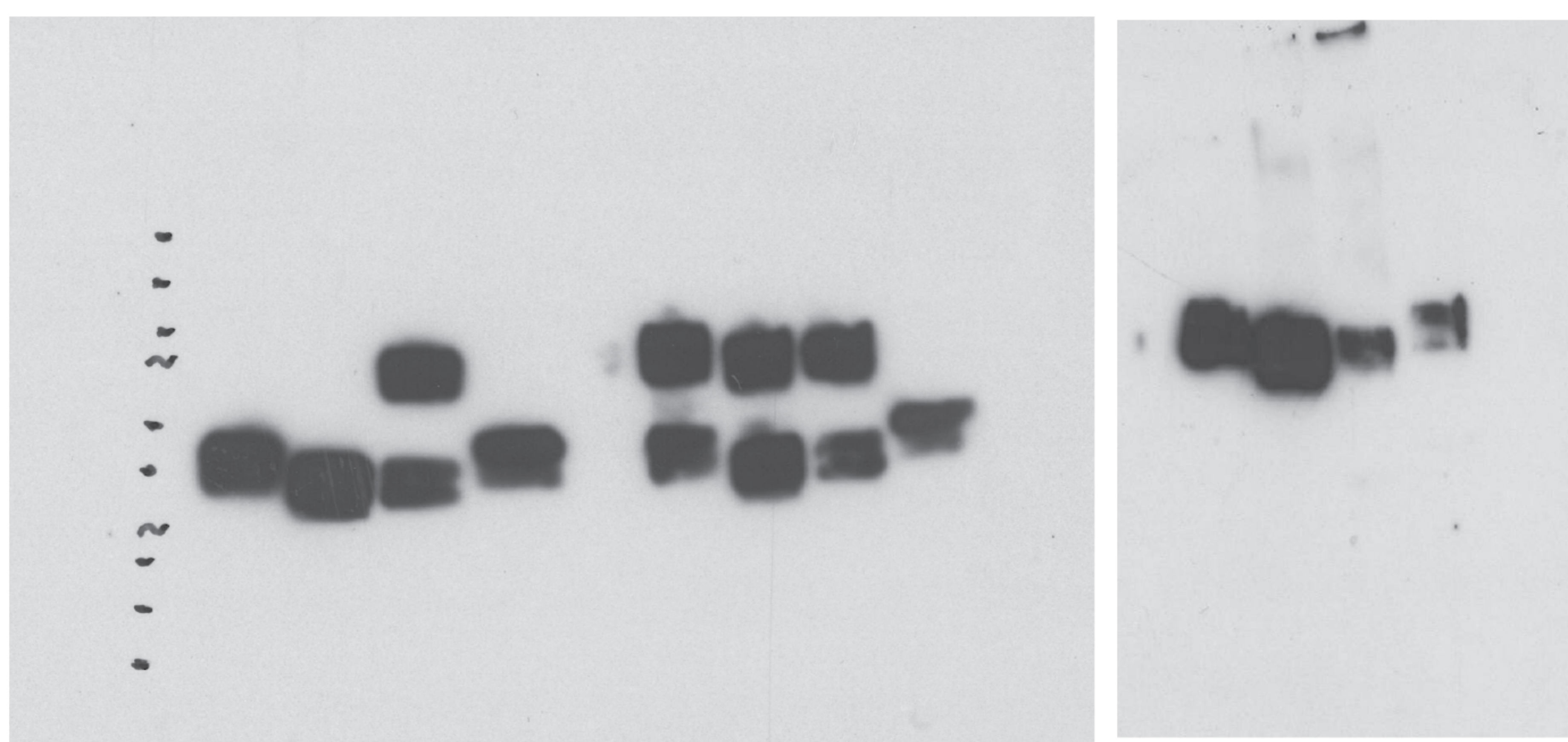

D

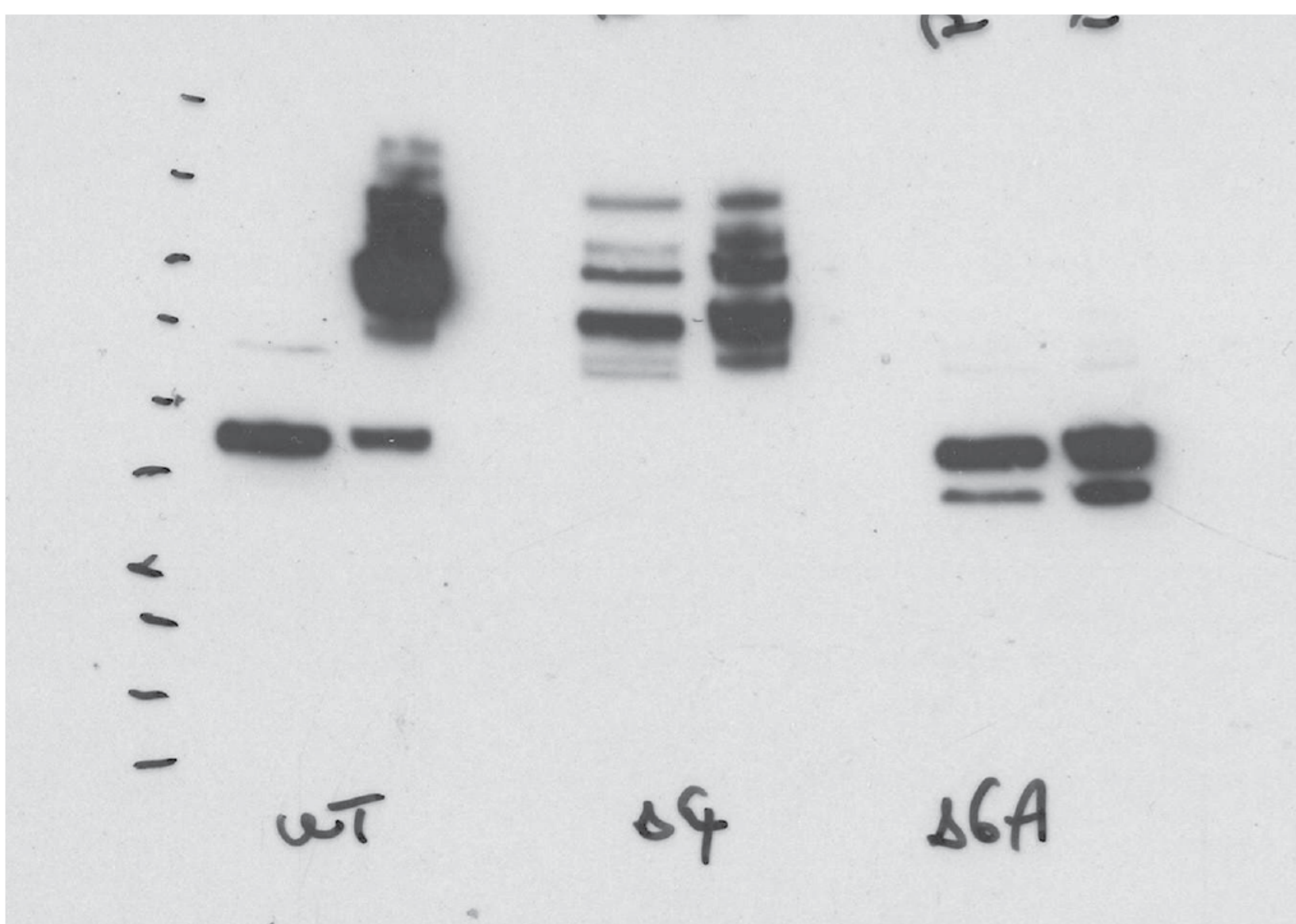

Figure 6 - source data

Supplement: Supplementary file 6 — Source Data for Figure 6 [file EMBJ-35-2484-s005.pdf]
